# Comparison of human and marmoset basic-level face categorization based on shape

**DOI:** 10.1038/s41598-025-31437-9

**Published:** 2025-12-17

**Authors:** You-Nah Jeon, Hector Y. H. Cho, Ashley C. Green, Elias B. Issa

**Affiliations:** 1 Zuckerman Mind Brain Behavior Institute, Columbia University, New York, USA; 2 Department of Neuroscience, Columbia University, New York, USA

**Keywords:** Neuroscience, Visual system, Object vision

## Abstract

Our ability to process faces depends on high-level visual areas in the brain which are thought to be partially shared between human and non-human primates. Nonetheless, studies of face recognition – whether for detecting or discriminating faces – in non-human primates have generally relied on 2D face photographs where low-level strategies relying on texture and appearance cues can be employed to adequately support task performance. Here, we tested humans and marmoset monkeys on a geometry-based invariant face category discrimination task under 3D pose and lighting variation using two textureless faces (human versus monkey) that only differed in their internal face geometry. Both humans and marmosets performed this shape based, basic-level face category discrimination task at comparable levels to their basic-level object category discrimination performance, while state-of-the art artificial vision systems specifically struggled on the face categorization task. Marmosets and humans both exhibited face-specific behavioral signatures including lower category discrimination performance for inverted faces, faces lit from below, and contrast reversed faces compared to normally lit upright faces. However, the magnitude of these deficits differed between marmosets and humans with marmosets less affected by inversion and more affected by contrast reversal than humans. Our results suggest that different primate species may differ in the details of their face processing. However, invariant face categorization ability based purely on shape appears to be generally preserved across simian primates from among the smallest to the most advanced, consistent with the presence of partially overlapping underlying high-level visual brain areas.

**Supplementary Information:**

The online version contains supplementary material available at 10.1038/s41598-025-31437-9.

## Introduction

Face processing is thought to depend on several hierarchically organized cortical areas in the ventral visual pathway^1,2^, but few studies have directly measured invariant face recognition behavior in monkeys to determine whether their visual system supports invariant face recognition behavior to the degree that it does object recognition behavior. Previous work on invariant object recognition behavior at the basic level (e.g. camel versus elephant discrimination; also known as core object recognition) emphasized the importance of testing the ability to recognize basic-level object categories across many identity-preserving transformations, including their position, scale (i.e. distance from the observer), background, and clutter^3–5^. Stimulus sets created with such variation have been used to compare different biological visual systems – human, macaque, marmoset – to each other and to artificial visual system^6–10^. Compared to these object recognition studies, face perception research in non-human primates has typically utilized photographs of faces or synthetic faces of either frontal views or of very limited number of views, of similar illumination, of similar size, and often without rich, varied backgrounds^11–20^. These stimuli, such as face photographs in the wild are seemingly natural but lack adequate independently controlled variation. Furthermore, the multiple cues that are present in these stimuli permit use of conflating low-level features, like texture and color, to solve the identification task and relatedly, obfuscate whether and what cortical mechanisms are necessary for robustly performing the behavior^21^. For example, various appearance cues such as hair or eye color in photographs can be easily picked up by simple visual systems but are not unique, are changeable, and may not generalize well to other views outside the frontal view.

Human face perception research has made a distinction between the role of shape and texture cues in face processing. Shape refers to the 3D geometry of the face, whereas appearance arises from a product of the pigmentation, illumination, and surface curvature^22^. Shape in particular is important for discriminating unfamiliar individuals across different views^23–32^. Inspired by these findings that shape and texture may tap into different recognition strategies and hence underlying mechanisms, we created a basic-level face category discrimination task – with textureless face images generated purely based on the geometry of the face – to rigorously compare monkey to human behavior on this general, arguably easy, aspect of face recognition where face geometries significantly differed by being drawn from two species. The two 3D face meshes were derived from a human and a macaque, respectively, in order to initially probe basic-level categorization ability (i.e., monkey versus human) as opposed to the subordinate level (i.e., individual A versus individual B)^33^. This mirrors our prior behavioral work in monkeys and humans examining basic-level object categorization behavior^10^. Moreover, compared to previous face recognition studies, we incorporated greater 3D identity-preserving transformations, namely face pose, lighting direction, and background variation. Remarkably, the resulting basic-level, species categorization face task was found to be much more challenging for artificial vision systems than a basic-level object categorization task, highlighting the specific challenge posed by the finer shape differences required for faces versus objects. In contrast to machines, we found that marmosets robustly performed the challenging basic-level face categorization at similar levels to their basic-level object categorization performance. Marmosets and humans displayed qualitatively similar deficits for inverted and contrast reversed faces, but they quantitatively diverged in their image-level patterns. Our results suggest that some general aspects of high-level face categorization ability may be shared across the simian primate clade, complementing the growing neuroscientific findings on the overall functional and structural homology of the ventral visual stream across marmosets, macaques, and humans^18,19,34,35^. Nonetheless, we observed quantitative discrepancies between humans and marmosets whose sources should ultimately be studied for understanding potentially differing strategies to face recognition across the simian primate clade.

## Results

### Humans and marmosets recognized face category defined by shape across changes in rotation, scale, lighting direction, and background

**Fig. 1:**
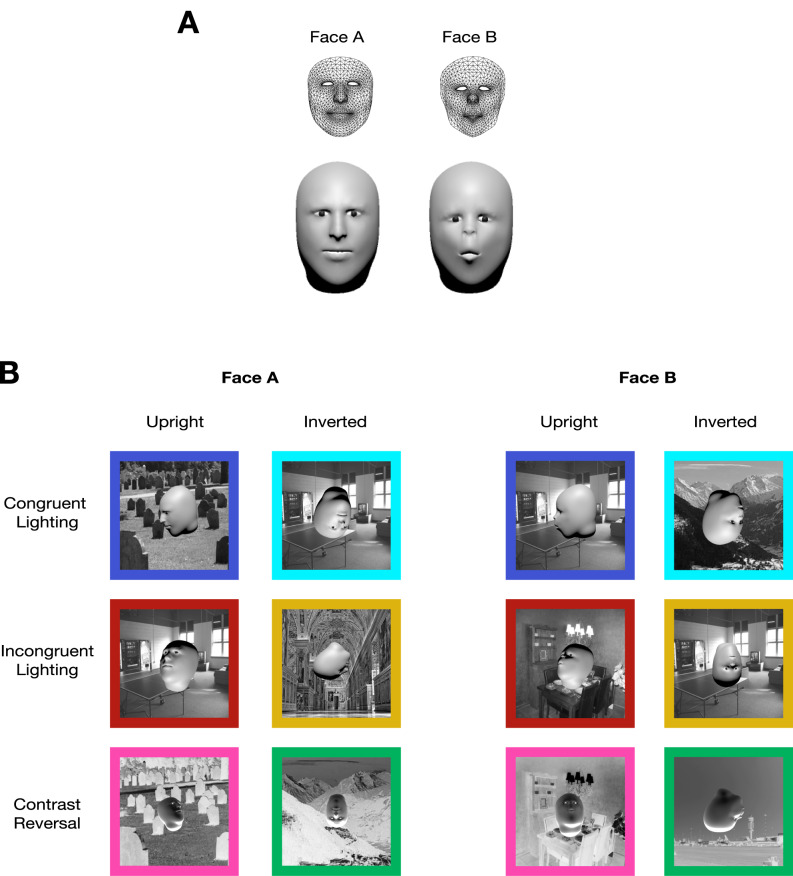
Face stimuli. (**A**) Human and monkey face. Original meshes acquired using Apple Face Capture depicted as wireframes and the output renderings from the final textureless 3D face models with eyes and head added. (**B**) Face orientation and lighting conditions. We combined two in-plane rotations, upright and inverted, and three lighting contexts, lighting from above, lighting from below, and contrast reversal, to create six different conditions (lighting direction is specified in world coordinates). Congruent lighting refers to the conditions where the lighting source is overhead (i.e., lit from below for inverted faces). An example image from each of the six different conditions is shown for both identities (25 images per condition used in the task).

**Fig. 2:**
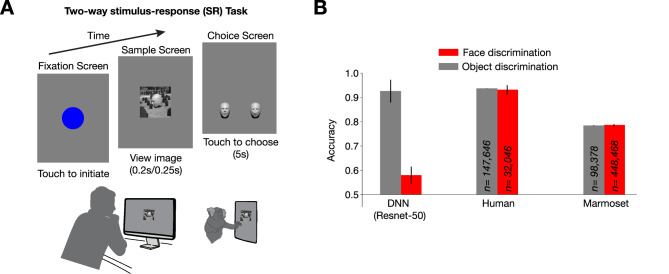
Face discrimination task and performance. (**A**) Task design. Both humans and marmosets performed a 2-way stimulus-response task where they chose which of the two faces a sample image corresponded to. The sample image was shown for 250 ms for marmosets and 200 ms for humans. (**B**) Task performance. Humans and marmosets far outperformed a DNN (ResNet-50) on the face category discrimination task. For comparison, performance numbers for the object category discrimination task (50 ms for humans and 250ms for marmosets) were adapted from Kell et al. Error bars indicate standard deviations across bootstrap resamples over trials for human and marmoset. Error bars for ResNet-50 are standard deviations across bootstrap resamples of training images.

We evaluated human and marmoset ability to discriminate two geometrically defined faces invariant to identity-preserving changes in rotation, scale, lighting direction and background. Our stimulus set included an equal number of upright and inverted faces rendered across three different lighting contexts: lighting from above (i.e. typical lighting), lighting from below, and contrast reversal (Fig. 1). Operationally, subjects, whether human or marmoset, participated in a two-alternative forced choice task (2AFC) where they chose which of the two faces was present in the flashed image (Fig. 2A; see Supplementary Video S1 of marmoset performing the 2AFC task). To benchmark the difficulty of this face category discrimination and ensure it was not solvable by low-level features, we used a state-of-the-art deep convolutional neural network (DNN) model, ResNet-50, pretrained on the ImageNet database. DNNs trained to categorize images broadly match human image categorization performance^36^ and are considered the leading models of human and macaque ventral visual cortex^6,37,38^. While an ImageNet-pretrained ResNet-50 achieved 92.5% on a variant of the 2AFC object category discrimination task previously studied in the lab^10^ comparable to human performance levels, the same model only managed 59% performance on the 2AFC face category discrimination task (chance = 50%), exhibiting a steep fall-off in performance from the basic-level object category discrimination task (Fig. 2B, core invariant object recognition; compare red to gray bars). This face task-specific drop in model performance occurred even though the object task images were rendered in a similar fashion to the face task images – both tasks utilized grayscale renderings from synthetic 3D meshes superimposed on random 2D natural image backgrounds. The poor basic-level face category discrimination task performance was not remedied by the number of face images used for training the linear SVM decoder nor by using a DNN encoder explicitly trained to identify faces from a large corpus of face photographs (VGG-Face) (Figure S1). The specifically face-trained VGG-Face network performed at 98.95% on face photographs but only 57% on our binary shape-based face category discrimination task^39^.

Far exceeding the performance of DNNs, humans performed our 3D face category discrimination task at 91% (pooled across 4 human subjects; individual subjects performed 88%, 97%, 94%, and 85%; see Methods for more details on human subject pools), and marmosets performed at 78% (Fig. 2B and 4 marmosets pooled; individual subjects performed 71%, 82%, 80%, and 76%; *n* = 1863 trials/session, Supplementary Figure S2). Notably, both humans and marmosets did not show any appreciable performance drop-off on the face discrimination task compared to their respective performance on the object discrimination task (Fig. 2B; compare red to gray bars). For all human and marmoset performance numbers, we applied a performance correction based on the lapse rate calculated as the subject’s error rate for the best image (selected on half of the trials and measured on the held-out half). Both humans and marmosets had a lapse rate of ~ 2%. This mild 2% adjustment allowed for quantitative comparisons of absolute visual ability (*sans* lapses in attention and/or downstream motor response errors during some trials). Thus, lapse-corrected primate performance allows direct comparison to DNNs which effectively have zero lapse rate as decoding of the face category was done directly from sensory representations in artificial vision models, without additional sources of attentional or motor noise as in the biological system.

### Face inversion and contrast reversal deficits in humans during shape based face category discrimination

**Fig. 3:**
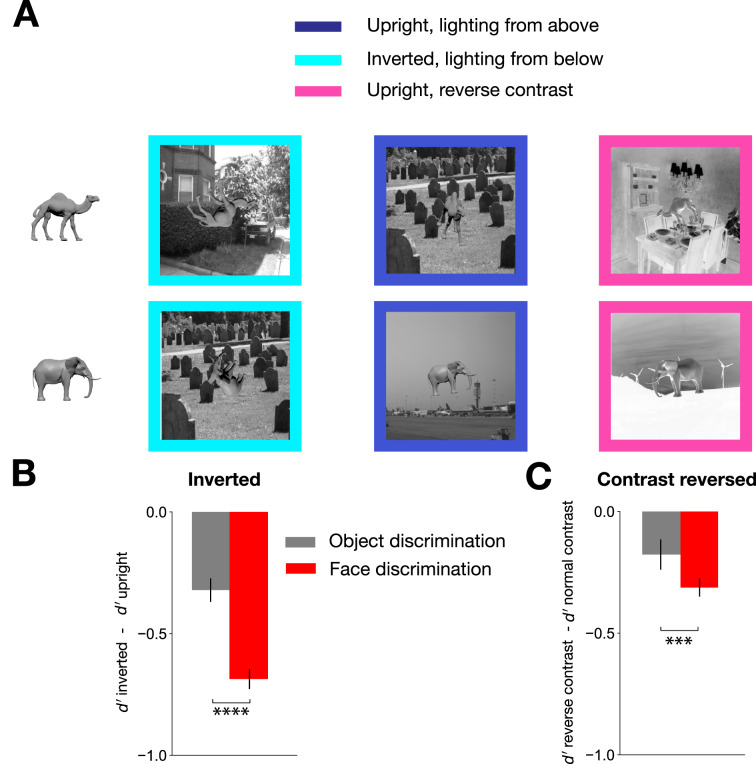
Control object category discrimination task and human performance comparison to face category discrimination task. (**A**) Object stimuli. Token camel and elephant images. Same stimulus generation process as in the face category discrimination task was used. An example image from the inverted, upright, and contrast reversed condition is shown in order. Upright/normal contrast condition only included lighting from above, inverted condition only included lighting from below, and contrast reversal condition only included upright objects in order to remove any interaction effect between conditions (lighting direction is in world coordinates). Each condition included 50 images in total, 25 from each object category. (**B**) Inversion effect. Humans showed a greater inversion effect for the faces than for the nonface objects. We randomly sampled 25 images from each context, upright and inverted, out of 50 images and calculated the difference of the average *d’* between the two. We repeated this process several times to obtain the error bars, which indicate 95% CI. (**C**) Reverse contrast effect. Humans showed a greater reverse contrast effect for the faces than for the objects. Error bars were calculated the same way as in (**B**). Here, we randomly sampled 25 images from reverse contrast and upright condition, respectively.

To test whether our basic-level face category discrimination task tapped into analogous mechanisms of canonical face processing, we assessed two well-known observations from human face recognition behavior: the inversion^40,41^ and contrast reversal effects^42,43^. Prior work had demonstrated these effects in a different task setting than ours, so we sought to determine whether inversion and contrast reversal effects manifested in performing an invariant shaped-based face categorization task. Importantly, the inversion effect has been shown not just in individual discrimination paradigms, but more broadly, in emotion, gender, and species discrimination paradigms^44,45^. Because the face inversion effect is meaningful when quantitatively larger than the size of the inversion effect for objects, we prepared a basic-level object category discrimination task for comparison to the face category discrimination task using the same distributions for each latent parameter – rotation, scale, lighting, and background (Fig. 3A). To emulate a species discrimination task, we chose camel and elephant meshes which, like faces, have similar stereotyped configurations, namely a head at the top, a body, and four legs below^46^. Again, to focus on discrimination based on geometric properties, we rendered the camel and elephant meshes without color and texture. When we compared performance for the inverted versus upright conditions in the two tasks (camel vs. elephant and monkey vs. human face), we found a disproportionately stronger decrease in inverted versus upright performance for the face categorization task compared to the object categorization task (two tailed t-test: t = − 14.30, p = $\:7.16\times\:{e}^{-17}$; Fig. 3B). Our tasks also revealed that contrast reversal induced a performance deficit in the face category discrimination task that was stronger than when contrast reversal was applied in the setting of shape-based object category discrimination (two tailed t-test: t = − 3.35, *p* = 0.0018; Fig. 3C). These results confirmed that our basic-level shape-based face category discrimination paradigm evoked face-specific visual processing deficits similar to prior work.

### Face inversion and contrast reversal deficits in marmosets

**Fig. 4:**
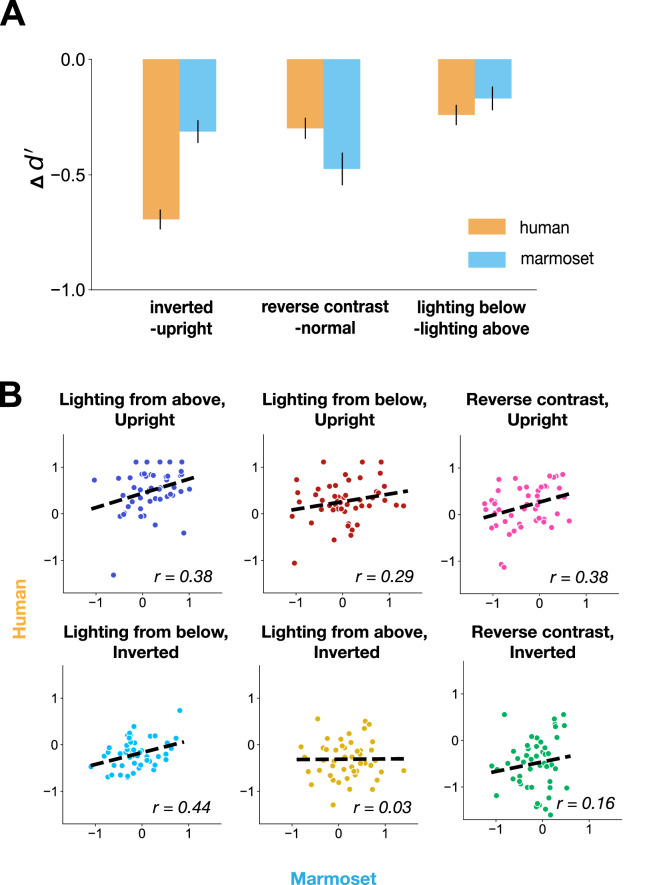
Human and marmoset image-by-image performance. (**A**) Comparison of the effect of different conditions in human and marmoset. Both human and marmoset subject pools showed deficits in discriminating faces that were inverted, contrast reversed, or lit from below. As in Fig. 3B, upright condition only included lighting from above, inverted condition only included lighting from below, and contrast reversal condition only included upright faces (lighting direction is in world coordinates). Each condition contained 50 images, 25 per species. Similar to Fig. 3B,C, the error bars indicate 95% CI, which were calculated by bootstrapping images in each condition. (**B**) *i1n* correlation per condition. *i1n* correlations were heterogeneous across conditions, with the contexts deviating from standard, natural viewing (lit from above and inverted; contrast reversed and inverted) having the smallest correlation (right panel in middle and bottom rows). All correlation-coefficients were noise-corrected. The top leftmost context, unright and downlit faces, is the “normal” condition (r = 0.38, p = 0.00644).

Like humans, marmosets were worse at recognizing faces that were inverted, contrast reversed, or lit from below compared to standard faces which were upright, normal contrast polarity, and naturally lit from above. The inversion effect was quantitatively stronger in humans than in marmosets, while the contrast reversal effect was quantitatively stronger in marmosets than in humans (Fig. 4A). It is important to note that our paradigm still exposed inversion effects in marmosets even though they were trained on both upright and inverted faces (see Methods), suggesting the overall robustness of face inversion deficits during basic-level face category discrimination. To check whether the inversion effect in marmosets was weaker for non-face objects than for faces, we re-analyzed our previously reported marmoset core (basic-level) object categorization dataset, specifically for upright versus inverted objects, and found that on the animal discrimination task used in our prior work (camel vs. rhinoceros), marmosets only showed a very mild inversion effect (∆d’_face, present work_ = −0.31 vs. ∆d’_object, prior work_ = -0.09; Supplementary Figure S3)^10^.

### Marmoset versus human behavior at the image level

To test whether marmosets matched humans at finer behavioral scales beyond absolute performance, we compared their relative image-by-image performance (vector of d’ per image or *i1n*, see Methods), which can expose differences and similarities across images that average task performance cannot^9^. We found a small but significantly greater than zero correlation across all 302 images (*r* = 0.23, p =$\:5.47\times\:{e}^{-5}$). When limiting to naturally rendered faces for more direct comparison to our prior object recognition work, the correlation was improved but still less than marmoset-to-human correlations in that prior object work (upright and lit from above faces, *r* = 0.38, *p* = 0.00643; inverted and lit from below, *r* = 0.44, *p* = 0.00139; first column of Fig. 4B compared to *r*_*object, prior work*_ ~ = 0.73). Conditions where the lighting context and the in-plane rotation created unnatural scenarios had the weakest marmoset-to-human correlation and thus the greatest divergence in image-level behaviors (*r* = 0.03, *p* = 0.836 for inverted, lit from above and *r* = 0.15, *p* = 0.298 for inverted, reverse contrast).

## Discussion

We tested humans’ and marmosets’ ability to discriminate textureless faces in a face species categorization task based on face geometry alone, a basic-level task that when benchmarked relative to our basic-level object categorization tasks was not nearly as easily performed by standard feedforward DNNs. On the other hand, we found that both marmosets and humans could robustly discriminate species when only given face shape cues, and that for these simian primates, their basic-level face categorization performance was, remarkably, at a similar level to their object categorization performance, unlike for artificial vision system controls. Moreover, humans and marmosets shared general face-specific perceptual deficits due to inversion, contrast reversal, and changes in the lighting direction. However, humans showed greater inversion effect while marmosets showed greater reverse contrast effect. While we only tested two faces of different species, our results provide a starting point for quantifying and understanding marmoset face recognition abilities and suggest that while shared qualitative perceptual deficits might be a reflection of conserved, underlying high-level visual cortical areas in simian primates^10,47,48^, the observed fine-grained differences, which could arise from distinct evolutionary adaptations but also require minimizing experimental differences to accurately compare between species, highlight the critical need to test multiple primate species on the same tasks to optimize comparative approaches and fully understand the diversity of face processing mechanisms across the primate clade.

### Species comparisons across visual face tasks

Our study indicates that humans and marmosets share coarse-level behavioral similarities in how well they categorize faces across contexts, but their behaviors can diverge at the image-level especially within certain lighting contexts (*r* = 0.38 and 0.44 for congruent lighting condition; *r* = 0.03 and 0.16 for incongruent lighting condition). This result is in contrast with our previous finding that marmosets are similar to both humans and macaques in their fine-grained patterns of core basic-level object categorization behavior patterns at the image-level (noise-corrected *r*_*object, prior work*_ ~ = 0.73)^10^. However, we note that our DNN baseline which does reasonably well in predicting human behavior on the object task in that prior work (prior object task: *r*_*DNN, human*_ ~ = 0.6) does remarkably poorly compared to marmosets in predicting human image-level patterns here in the face task (upright, congruent lighting condition: *r*_*DNN, human*_ ~ = 0.01 versus *r*_*marmoset, human*_ = 0.38). The higher DNN model-to-human correlation in the prior object task compared to the present face task could be driven by the fact that the prior object categorization task was not purely a geometry based task – that prior task included texture information where models may excel like humans and was more weighted to contrast and position variation compared to our face task which fixed face position at the fovea and was weighted toward lighting and pose variation. Alternatively, face perception may be a more recent visual faculty, may have consequently had differing evolutionary pressures for humans as opposed to monkeys, and may not be as shared as core basic-level object recognition across simian primates with differing social niches. Experiments probing the marmoset-human comparison on face recognition in greater detail could help in explaining the significant difference between the results from the present face task and our previous object task: (1) running each lighting/appearance context as a separate task more like prior work in object recognition to ensure a consistent task strategy (2) compare the image-level patterns across monkeys and humans for the textureless object recognition task used here as a control, and (3) compare face recognition signatures on conspecific (marmoset faces) individuation versus the species discrimination task used here.

Previous studies have reported mixed results with regards to the species-specificity of inversion effects^49,50^. Chimpanzees exhibited the inversion effect for both human and chimpanzee faces^17^. Capuchins also showed the inversion effect for both conspecific and human faces^51^, while squirrel monkeys showed an inversion effect only for human faces but not monkey faces (both conspecific and heterospecific)^16^. Rhesus macaques showed an inversion effect for human faces but not macaque faces^15^. To compare with these studies, it will be necessary to test marmosets on marmoset faces in addition to human faces. Whether or not image-level patterns match better for the conspecific faces across species needs to be investigated. Thus far, we have shown that marmosets display an inversion effect for both human and macaque faces. We note that different task paradigms are used across studies (e.g., preference measured by eye movements vs. forced choice task), which may need to be controlled to limit potential differences across studies. We also note that the inversion effect has been reported in non-identity discriminations such as discriminating the emotion or species of the face (as in the present study). Chimpanzees showed an inversion effect in discriminating different primate species from faces^44^, while humans showed an inversion effect in discriminating emotions from faces^45^. Future research can test whether the inversion effect is stronger for individual recognition than species recognition by using faces from the same species and testing whether this leads to a better matching inversion effect between humans and marmosets.

### Marmosets as a model organism to study geometric face perception

Lingering doubts about the marmoset’s visual face recognition ability stemmed from their natural arboreal habitat—the tree canopy in the Amazonian rainforest—where visual access may be hindered by the thick foliage^52^. Moreover, their rich vocal repertoire is frequently suggested to be a primary means of social interaction, including identifying conspecifics in such a natural, visually occluded habitat^53–56^. Nonetheless, in keeping with the ethological perspective, it is also important to consider that marmosets maintain tight bonds with their partners^57^, use various facial expressions^58^, and engage in cooperative breeding^59^, activities that require close physical distance, close enough for visual face recognition. In lab settings they preferentially look at conspecific faces in photographs and use the gaze direction of other individuals to locate food^60,61^. These observations point to a possibility that marmosets are capable of identifying individuals via visual face recognition. Still, another reason for lingering skepticism is whether such a small simian primate brain could support face discrimination in computationally challenging human-level tasks^62,63^. Building upon our previous work showing that object recognition in marmosets is a surprisingly good match to that in macaques and humans, the present study rigorously tested marmosets’ ability to distinguish faces in a face category discrimination task that was more computationally challenging than prior object category discriminations. Indeed, we intentionally chose to use a human face to test the limits of marmoset visual aptitude in performing similar tasks as humans. Our study provides critical behavioral evidence affirming that marmosets are a suitable candidate to serve as small animal model organisms to study the neuroscience of shape-based face perception.

### Distinct roles of texture and shape in face processing

Different aspects of face recognition are influenced to different degrees by texture and shape. Texture seems to be the dominant cue for recognizing familiar faces, while both texture and shape cues are important for discriminating unfamiliar individuals^23–28^. In addition to familiar face recognition, pigmentation-based face discrimination, but not shape-based discrimination, is selectively impaired by contrast negation^29^. On the other hand, shape plays a critical role when learning a new identity or generalizing to different views^64–67^. Our study probed the marmoset’s ability to discriminate shape defined textureless faces with a particular emphasis on doing so across viewpoints and lighting. Future studies could test the complementary role of texture cues in an invariant fine face discrimination task across viewpoints and lighting, including examining the strength of inversion and contrast reversal effects when adding texture cues. For example, does adding texture significantly improve performance across viewpoints, and does contrast reversal lead to bigger performance decrements when discriminating between textured faces? As face tasks expand beyond identification, we anticipate an interplay between the type of task and the specific facial features that are emphasized. For example, previous studies found that chimpanzees relied on color more than shape to discriminate the age of conspecifics while Japanese macaques used facial morphology to discriminate the sex of conspecifics^68,69^. Future work can test whether or not marmosets exhibit particular dependence on either shape or texture cues for various forms of inference in faces, and if this dependence on either cue is specific to conspecific faces.

### Fine 3D geometry discrimination exposes gap between biological and artificial systems

Our novel, geometric face category discrimination task for basic-level face distinctions challenged a state-of-the-art ImageNet-pretrained DNN in a way that the analogous basic-level object categorization task did not (Fig. 2B). In prior work, the basic-level object categorization task was made more difficult by including object position variation to thwart low-level features. Here, without requiring any position variation at all (all faces are presented centrally in the image, effectively at the fovea), we found that our task was still computationally challenging not just for low-level control features in early, high dimensional layers but was also challenging for the best layers in the network as a whole. The difficulty inherent to our face task was created by naturally varying 3D pose and lighting rather than by using large 2D position shifts to different parts of the visual field to thwart early, retinotopic model layers. Overcoming local 3D variations to reliably detect geometry differences proved challenging even for high-level features in artificial systems. Indeed, when we reduced the strict geometry demands of our face task, the DNN tested (ResNet-50) benefitted in task performance from adding even the most minimal pigmentation–no hair, just adding skin color and moderate texture (Figure S4). Future studies can investigate if making artificial systems more shape-sensitive, either by training them on similar stimuli to ours, or optimizing for reconstructing the underlying 3D meshes such as in depth estimation tasks^70,71^, can make them perform better on the shaped-based basic-level face category discrimination task and whether, as they perform better on the task, they also go on to exhibit similar image-level patterns as humans and marmosets. In this virtuous experiment-modeling loop, insights from high-level face recognition behavior, where primates have evolved to excel, can help bridge the remaining gaps between biological and artificial vision. Importantly, to complement the behavioral phenomena in constraining models, more research will be needed to investigate the underlying neural mechanisms and accompanying computational principles driving shape-based face discrimination across simian primates.

## Methods

### Stimuli

#### Face stimuli

We created two faces for the basic-level face category discrimination task—human versus monkey—termed Face A and Face B by utilizing Apple’s face tracking capability in iOS devices (see captured meshes in Fig. 1A). The TrueDepth camera in Apple iPhones estimates the relative depth of points projected on a face. Developers can access the coordinates of the imaged 3D face mesh along with blendshapes—coefficients representing the detected facial expressions via movements of the estimated 1220 vertices—and other information through the Apple ARKit API (https://developer.apple.com/documentation/arkit/arkit_in_ios/content_anchors/tracking_and_visualizing_faces)*.* For acquiring the particular face meshes used in this study, we built upon FaceCaptureX, an iPhone application written by Elisha Huang^72^, which returns the 3D mesh coordinates of the face currently in view along with the blendshapes and photos of the face at a given framerate. We edited the source code of the application to collect extra information including the projective transform and the resolution of the captured photos. After collecting a face mesh using the customized version of FaceCaptureX, the mesh was stitched onto a faceless, dummy 3D head model to generate the full 3D face plus head object using custom code written in MATLAB (code available at https://github.com/issalab/Jeon-apple_facestim_generation)*.* The stitched 3D face mesh plus head mesh was then exported to the open-source Blender software for mesh editing (http://www.blender.org)*.* Eyes were added to the full 3D face mesh, and the entire mesh was smoothed using the *Shade Smooth* function in Blender, which locally interpolates vertex normals. Critically, we added identical head and eye meshes to face meshes A and B from both identities to minimize the difference between them the two identities other than their internal face geometry.

Face A was modeled after one of the lab members (40 yo male) and Face B was modeled after a rhesus macaque (6 yo male), both with neutral expressions. Ultimately, we chose face meshes from different primate species to create a greater separation of face geometry. While this species geometry discrimination task might be easier, the task provided a good starting point for probing marmoset basic-level face category recognition ability and proved to be surprisingly challenging for DNN model controls. Human behavior data on this task showed that the inversion effect was comparable in magnitude across Face A and Face B (t = 0.898, p-val = 0.375; Supplementary Figure S5), suggesting that in this species discrimination setting faces of both species engaged the signature aspects of face processing.

From each face mesh, we rendered 151 images generated using different 3D viewing parameters (images available at https://github.com/issalab/Jeon-et-al-Marmoset-Face-Behavior and see Figure S6). To emulate real-life interactions with faces where humans usually foveate on an individual’s face via centering eye movements, we consistently placed the 3D foreground face centrally on top of a randomly chosen natural image background. Ten backgrounds were randomly selected from an open-source database and used across both face categories such that the background image identity provided no information about foreground face category^73^. In each image, the face was randomly scaled between ~ 4.5° to 9, and rotated horizontally (− 90° to + 90°) and vertically (− 45° to + 45°). All three parameters – size, horizontal rotation, and vertical rotation – were drawn randomly from the uniform distribution of their respective range. While previous research that tested object recognition in marmosets used a greater range of views for objects – − 180° to + 180° in all three axes – we removed the extreme poses for faces here to ensure that most of the internal facial features were visible, as opposed to viewing the back of the head. Next, we added essentially three different lighting contexts where the faces were either lit from above (i.e. normal lighting), lit from below, or contrast reversed. In each lighting condition, the faces were presented either upright or inverted. In total, there were 6 different contexts (three lighting contexts by two in-plane rotations) with 25 images per context. In the reverse contrast condition, both the face and the background were contrast-reversed to prevent performance from being affected by the ease of foreground-background segmentation. For Figs. 3 and 4, upright condition included only the “true” upright condition, where the faces are upright and lit from above. Inverted condition only included the true inverted condition, where the faces are inverted and lit from below. The reverse contrast condition only included the upright faces, in order to remove any interaction effect between the lighting context and in-plane rotation context. These three conditions are colored navy, cyan, and pink, respectively in Fig. 1. In addition to these 150 rendered images per face mesh where the face was overlaid on a background, we included a token image with no background but containing an upright, downlit face in the frontal view (0^o^ rotation) presented at ~ 9° size. The entire rendered image including the background subtended ~ 20° in the marmoset touchscreen setup in their homecage (where the marmoset head was generally positioned near the juice reward tube at a fixed distance from the screen). For humans, who performed trials on a computer, assuming typical ergonomic arrangements, we estimate that the images (face plus background) were presented at 4°–12° in visual angle.

For Supplementary Figure S4, textured face meshes were created using one of the photos taken from the FaceCaptureX application. A photo was selected and edited in Photoshop to remove non-face areas of the image so that only the texture of the face area remained, and the outside was colored in a similar color as the skin of the face. This photo was then used as a texture map for the 3D face mesh. Like the textureless meshes, the textured meshes were stitched to the head mesh in MATLAB and the full face plus head mesh was exported to Blender for mesh editing. Eyes were added, the entire mesh smoothed, and the texture map applied in Blender.

All stimuli were generated using MkTurk, a web-based behavioral platform developed in the lab (*mkturk.com/landing*; code available at https://github.com/issalab/mkturk)*.* See Methods in Kell et al. 2023 for further details about MkTurk. In addition to presenting 2D images, MkTurk is equipped with 3D scene creation capability using the open source ThreeJS library for rendering in WebGL (www.threejs.org)*.* Experimenters can specify scene parameters, including all the viewing parameters listed above to modify 3D object renderings upon every displayed frame using the real-time web-based 3D rendering engine. All rendered image stimuli are shown in Supplementary Figure S6.

#### Object stimuli

Two object classes – camel and elephant – were chosen for the control object discrimination task. As in the face discrimination task, these categories reflected a species discrimination task between two animals that are relatively similar in general layout – four legged, similar body plan – but different in the exact geometry. A 3D object model for each animal class was downloaded from the SketchFab library (https://sketchfab.com/)*.* Both objects were rendered in the same gray color. We generated 151 images of each animal class using the same generative distribution of viewing parameters and applied the same contexts as in the face stimuli (including lighting direction changes, contrast reversal and inversion). For the canonical token image, we chose a side view of an upright camel and elephant, as compared to the frontal view for faces. All rendered image stimuli are shown in Figure S7.

### Two alternative forced choice stimulus-response task

Humans and marmosets participated in the face discrimination task, and humans were tested on the follow-up object discrimination task. Subjects initiated a trial by touching a dot at the center of the screen. A sample image was then flashed (250 ms for marmosets and 200 ms for humans). After the sample image disappeared, two token images appeared that were representative of the two choice categories, and the subjects chose which of the two species was seen in the sample image. The choice screen was always fixed such that the token image of Face A appeared on the left, and Face B on the right, making this a stimulus-response task (i.e. always choose left for Face A). For the object discrimination task, camel always appeared on the left and elephant on the right.

### Subjects

Four common marmosets (*Callithrix jacchus*; subjects J, G, V, and S) were tested on the same basic-level face category discrimination task. All animals used were bred and housed in a marmoset research colony. All procedures were in compliance with the NIH guidelines and the Columbia University Institutional Animal Care and Use Committee (IACUC). All experimental protocols were in compliance with the NIH guidelines and standards of the American Physiological Society, and approved by the Columbia University Institutional Animal Care and Use Committee (IACUC). Human behavioral data were collected from the Amazon Mechanical Turk platform (MTurk), where subjects participated either in the basic-level face or object category discrimination paradigm described above. In total, 68 human subjects participated in the study and informed consent was obtained from all subjects. All data were collected in accordance with the Institutional Review Board of Columbia University Medical Center.

### Marmoset behavior

Marmoset behavioral data was collected in their homecage, where an individual monkey performed trials on a touchscreen device (Google Pixel C tablet or Google Pixel 4xl phone). Marmosets in their homecage participated in the task about 3 h per day for a juice reward. For testing on the full task, monkey J, G, and V completed ~ 1800 trials per day for ~ 3 months. Monkey S completed around 600 trials per day. In total, Monkey J finished 106,717 trials, Monkey V 189,988 trials, Monkey G 149,438 trials, and Monkey S 2,843 trials. Subjects were given up to 5 s to make a choice, and all data presented in this paper, both individual and pooled, used trials where the monkeys made a choice (left or right) between the two face categories within the five second choice timeout period (see Supplementary Video S1). All four monkeys went through a series of training stages. For the first stage, they discriminated between the token images with no pose variation and no backgrounds. We slowly added more variations such as size, pose, position, and background. In the final stage of training, the marmosets were introduced to the high-variation images that were similar in complexity to the actual test images. Once the monkeys saturated their performance on training images, we switched them to the testing image set for the basic-level face category discrimination task. Lapse rate for the marmosets was calculated by partitioning the trials into two halves, finding the best-performing image in one half, and calculating the cross-validated performance for that image in the other half of trials. For all main analyses of the paper, we pooled trials across the four marmoset subjects.

### Human behavior

For the face discrimination task, 37 subjects were recruited to complete ~ 500 trials per subject. Seven of them were removed from the subsequent data analysis because they either performed near chance (0.5) or completed fewer than 20 trials. While Amazon MTurk provided a convenient way to amass a large number of psychophysics trials, it can be uncontrolled relative to a lab setting and thus, might include subjects that are less motivated and focused. A large performance spread on the face recognition task in the 30 subjects (76.57 ± 12.09%) indicated potential non-perceptual variability in task performance. Thus, we recruited 4 out of the 30 subjects to collect a larger number of trials per subject (8,011.5 ± 418.9 trials), more on par with the number of trials per marmoset subject. Indeed, the 4 subjects performed much better than the 30 subjects, at around 91% (Fig. 2B). A much larger number of trials from a smaller number of subjects also enabled calculating a more reliable image-by-image difficulty in the basic-level face category discrimination task (i.e., higher internal consistency of data on *i1n*, see Methods “quantification metrics and statistical analysis”). We pooled trials across the 4 subjects for all analyses. Lapse rate for the face discrimination task was calculated in the same way as for marmosets. For Fig. 1D, we bootstrap resampled trials from the 4 subjects 1000 times and reported the mean and the standard deviation of the performance. For the object recognition task, 31 subjects were recruited to complete ~ 500 trials per subject. Four of them were removed from the data analysis following the same exclusion criteria as in the basic-level face category discrimination, pooling trials across the remaining 27 subjects for all analyses.

### DNN model behavior

We used the off-the-shelf Resnet-50^74^ and VGG-face^39^ pretrained, convolutional feedforward models for DNN model controls. Resnet-50 is a state-of-the-art deep net trained on the ImageNet dataset and excels in object recognition from natural images. By contrast, VGG-face is specifically trained on a large-scale human face dataset to individuate identities across a large corpus of face photographs. For each model, we trained linear classifiers on the activations from the penultimate layers and reported the classifier performance (see Kell et al., 2023 for details). To train linear decoders, we created a training dataset that was distinct from the actual test set but used similar distributions for latent variables – rotation, size, lighting, and background – as the test set for measuring human and marmoset behavior. We used the same uniform distribution between ~ 4.5° to 9° for size, -90° to + 90° for horizontal rotation, and − 45° to + 45° for vertical rotation. For lighting, we randomly varied the lighting direction horizontally from − 1 to 1 (left to right), and vertically from − 1 to 1 (below to above) in order to expose the models to more lighting variations than there exist in the test set, which only included two lighting directions (directly above or below). We did not include reverse contrast images in the training since this condition is not part of our regular experiences with faces. We ran the images of the training and test sets through the network, and obtained the activations from the penultimate layer of that network (2048 activation units per image). We trained linear classifiers with these activations for different numbers of images in this training set. We randomly sampled different training images 1000 times for training linear support vector machines (SVM) with L_2_ regularization and a hinge loss. For the main figures in the paper, we chose the number of images that saturated performance on the test set (approximately 800 images per class, see Supplementary Figure S1). Model performance was reported as the mean and standard deviation of the classifier performance over the 1000 iterations of resampling images for classifier training. For each image, d’ was calculated as the distance from the hyperplane learned by a linear SVM.

### Behavioral performance metrics and statistical analysis

For each individual image *j*, behavioral *d’* of primates was calculated as follows:

1 $${\mathrm{D}}^{\prime}_{{{j}}}={\mathrm{z}}({\mathrm{HR}}_{{{j}}})-{\mathrm{z}}({\mathrm{FA}}_{{\mathrm{j}}})$$

where *HR*_*j*_ and *FA*_*j*_ are the hit rate and the false alarm rate – proportion of trials where the image *j* was correctly classified and the proportion of trials where any image was incorrectly classified as the object category that the image *j* belonged to, respectively. *i1* is a vector of d’ for all the images for a given discrimination task. Thus in this study, *i1* is a 302-length vector that contains a *d’* estimate for the 151 images from each of the 2 classes. The normalized *i1*, or *i1n*, was calculated by subtracting the mean performance for the correct category (average d’ across 151 images) from each d’_*j*_ to remove any mean difference in difficulty between the two classes, and the figures and texts refer to this category mean subtracted *d’* as *i1n*. After calculating *i1n* of the pooled human and pooled marmosets, these image-by-image performance patterns were compared against each other by calculating the noise-corrected Pearson’s correlation coefficient. The coefficient was calculated by randomly partitioning the trials into two halves, computing the *i1n* for each half, and calculating the mean of the correlation of the human and marmoset *i1n* across the two split halves. We normalized this split-half correlation by the internal consistency of *i1n* within each species. The internal consistency was calculated as the geometric mean of the correlation coefficients of the *i1ns* across the two trial split halves within each species (to provide a ceiling correlation given trial-by-trial noise in the data).

2 $$\:{R}_{nc}=\:\frac{\frac{1}{2}\left(r\right({V}_{a0},\:{V}_{b1})\:+\:r({V}_{a1},\:{V}_{b0}\left)\right)}{\sqrt{r({V}_{a0},\:{V}_{a1})+r({V}_{b0},\:{V}_{b1})}}$$

where r() calculates the Pearson’s correlation coefficient, and $\:{V}_{a0},\:{V}_{a1}$ refers to two split-halves of a system *a*, and $\:{V}_{b0},\:{V}_{b1}$ to two split-halves of a system *b*. This split-halves noise ceiling was used to normalize raw correlation values to produce the noise-corrected correlations reported in Fig. 4B.

## Supplementary Information

Below is the link to the electronic supplementary material.

Supplementary Material 1

Supplementary Material 2

## Data Availability

All face and object images used in the study are available at the following url: https://github.com/issalab/Jeon-et-al-Marmoset-Face-Behavior/tree/main.
