# Supplementary material for: Comparison of human and marmoset basic-level face categorization based on shape
Source: Sci Rep. 2025 Dec 17;16:1785. doi: 10.1038/s41598-025-31437-9 (PMC12804965; doi:10.1038/s41598-025-31437-9)
Supplement: Supplementary file 1 — Supplementary Material 1 [file 41598_2025_31437_MOESM1_ESM.pdf]

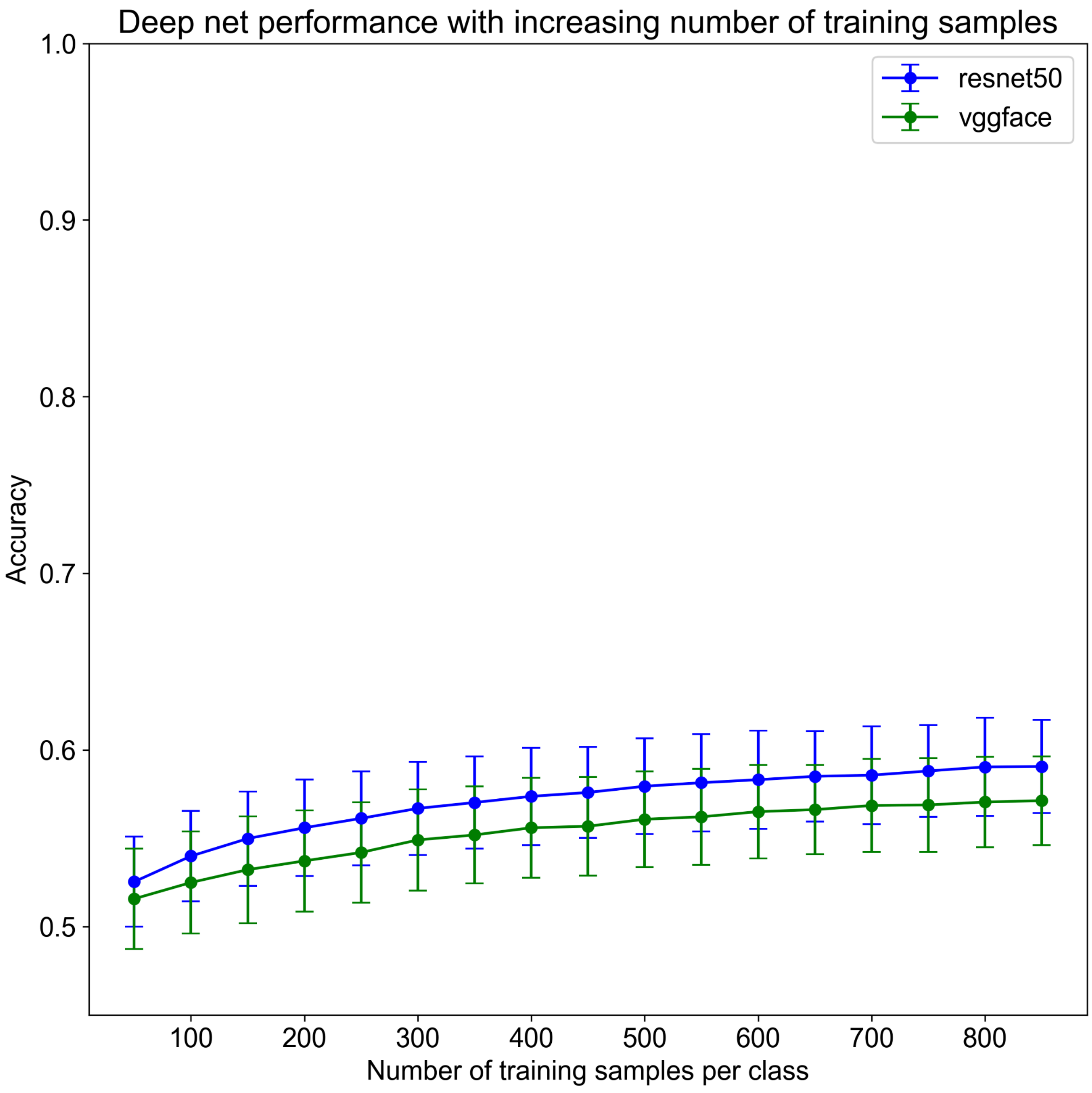

Figure S1. DNN performance on the face discrimination task as a function of number of training images. In order to ensure that we reached a performance plateau with the deep net controls, we trained linear decoders with different numbers of images per identity in 100 image increments. Bootstrapped error bars are standard deviations over 1000 classifiers trained on random samples of training images. The performance number from using 800 images/identity in classifier training was used in **Figure 2B** in the Main Text.

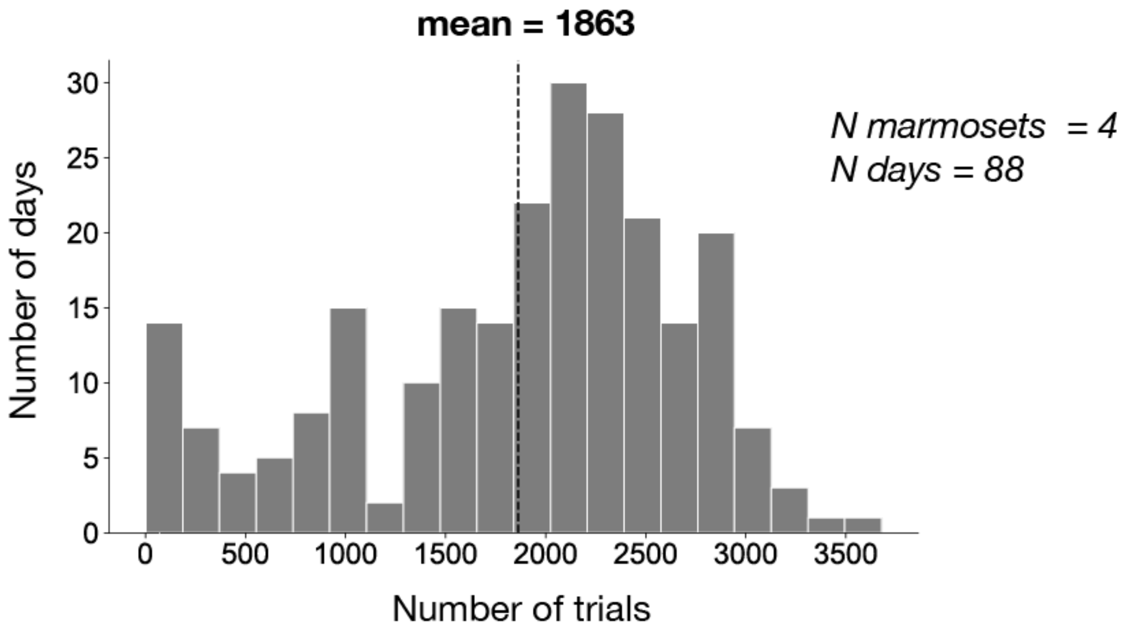

Figure S2. Number of trials performed per day by marmosets. Three monkeys were tested over a period of 2-3 months. The fourth monkey was tested over a period of a week.

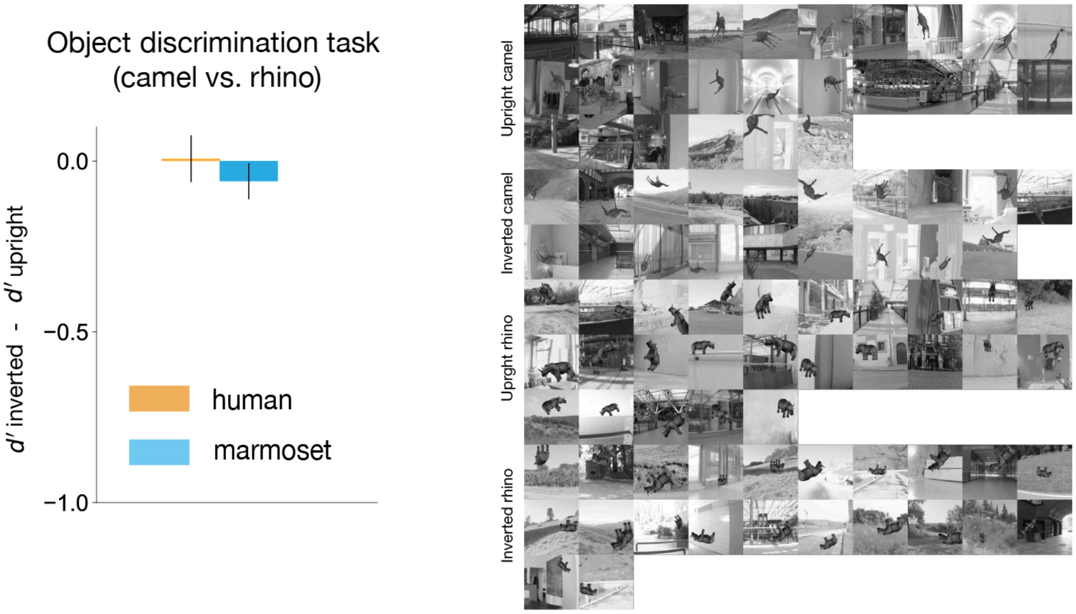

Figure S3. Inversion effect in an object discrimination task (camel vs. rhino). Re-analyzing data from Kell et al. 2023, we found that marmosets suffered a very mild inversion effect (Δd' < -0.1) in a camel vs. rhino discrimination task. In this task, 100 images from each object category were presented to subjects. Among these, 26 images of upright camel, 19 images of inverted camel, 25 images of upright rhino, and 22 images of inverted rhino were chosen. We randomly sampled 20 images from each condition, upright and inverted, and calculated the difference of the average *d'* between the two. We repeated this process 50 times. Error bars indicate 95% CI over the 50 repeats. All stimuli used in the analysis are shown on the right.

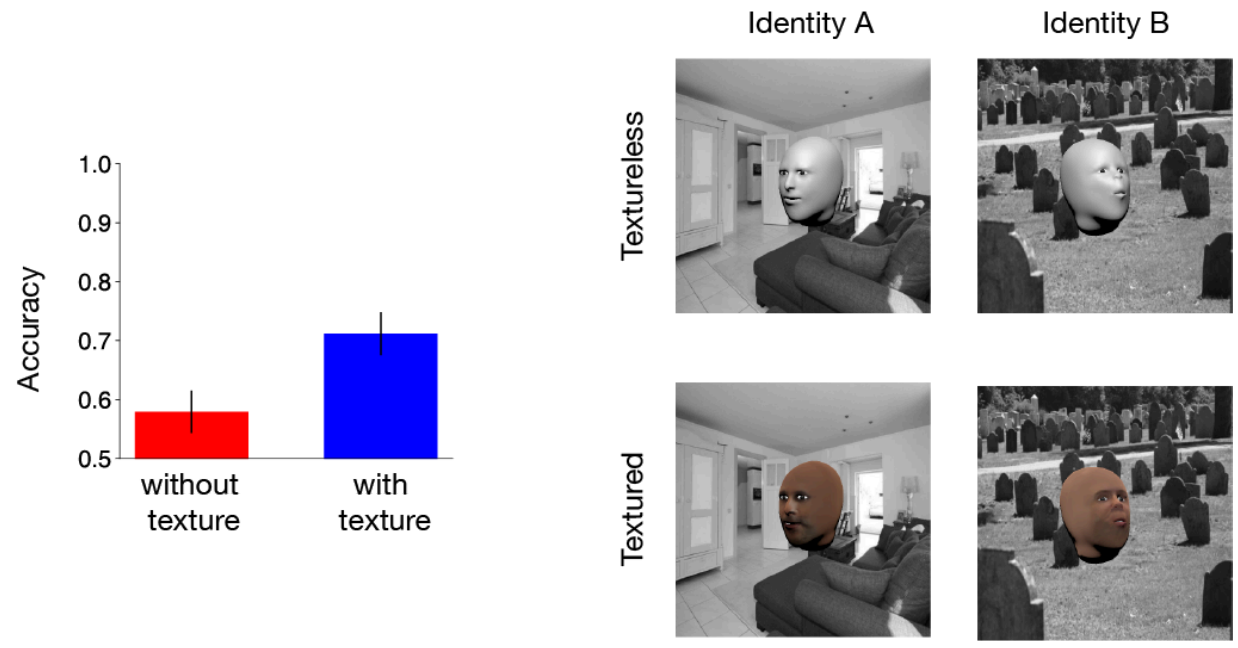

Figure S4. Comparison of ResNet-50 performance for textured vs. textureless face discrimination. When texture is added to the face, model accuracy on face discrimination significantly improves. An example image from textureless and textured versions of each face identity is shown (right panel).

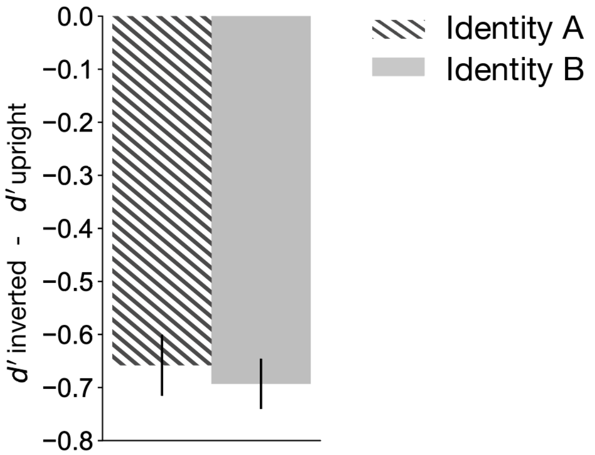

Figure S5. Comparison of inversion effect for two identities. In order to establish that both identities evoked face-specific perceptual effects, and at a comparable level, we quantified the inversion effect in human subjects per identity and found that both Identity A and Identity B induced a similar level of inversion effect.

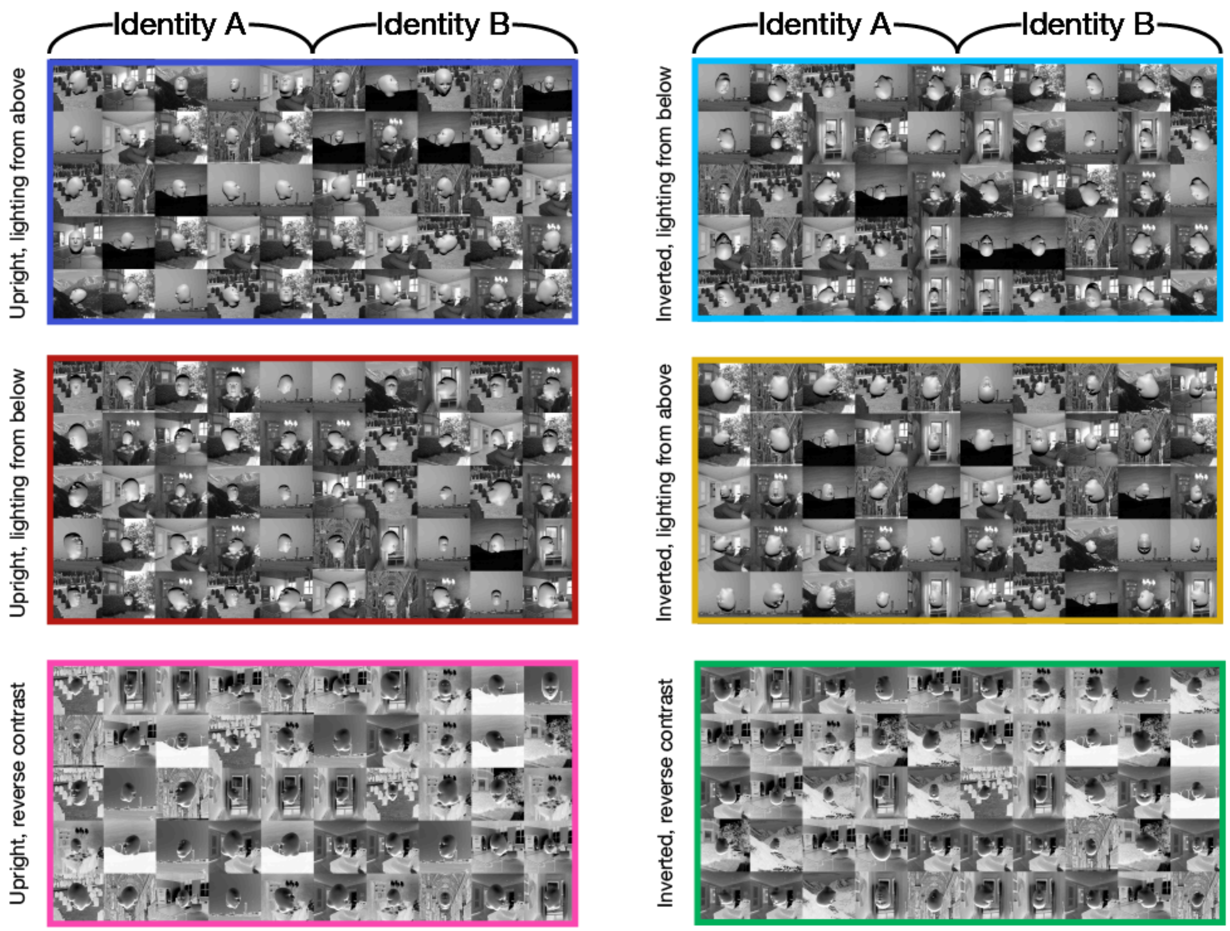

Figure S6. All 300 images used in the shape based face discrimination task. Different colors indicate different viewing contexts, as in Main Text **Figure 1B**.

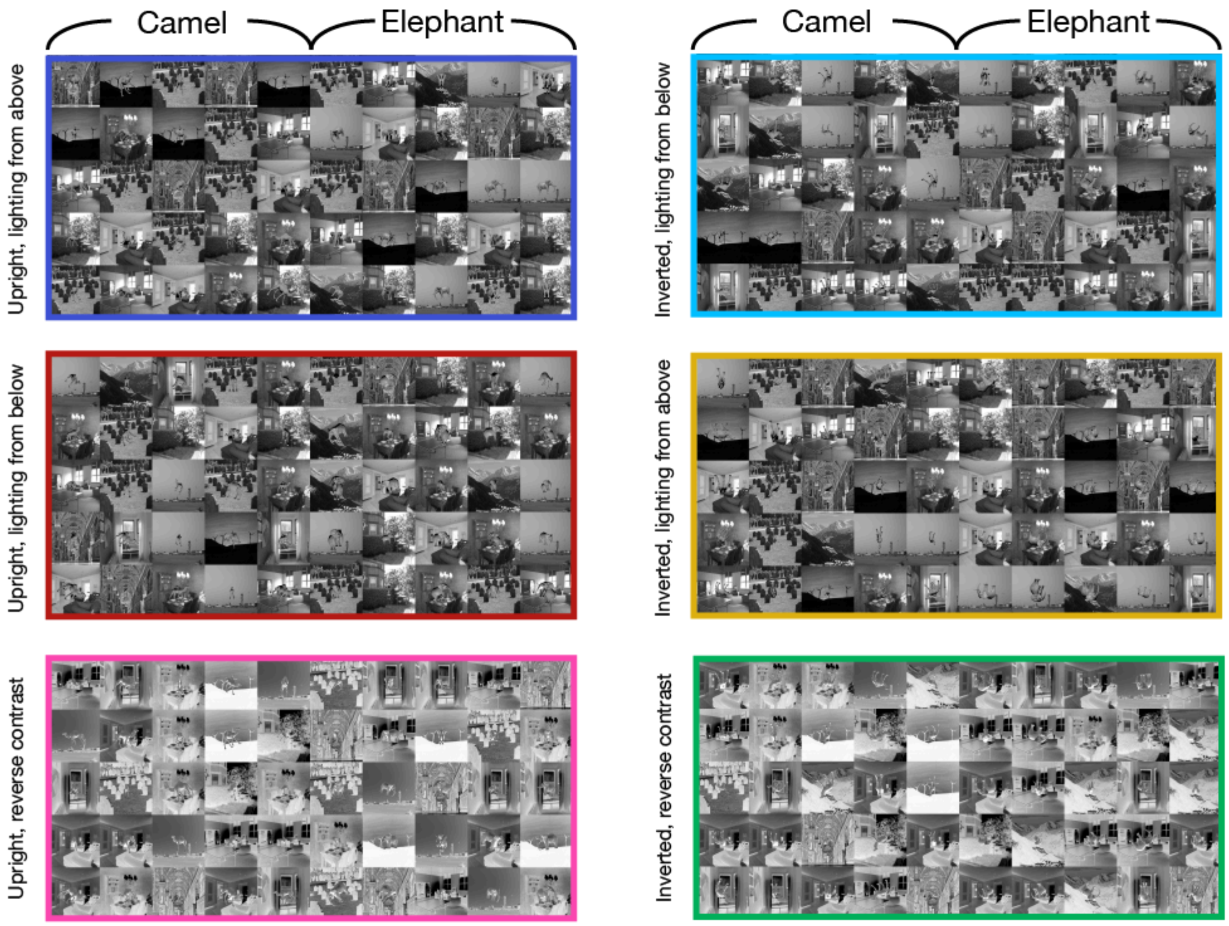

Figure S7. All 300 images used in the shape based object discrimination task (camel vs. elephant). Different colors indicate different viewing contexts, as in Main Text **Figure 1B**.

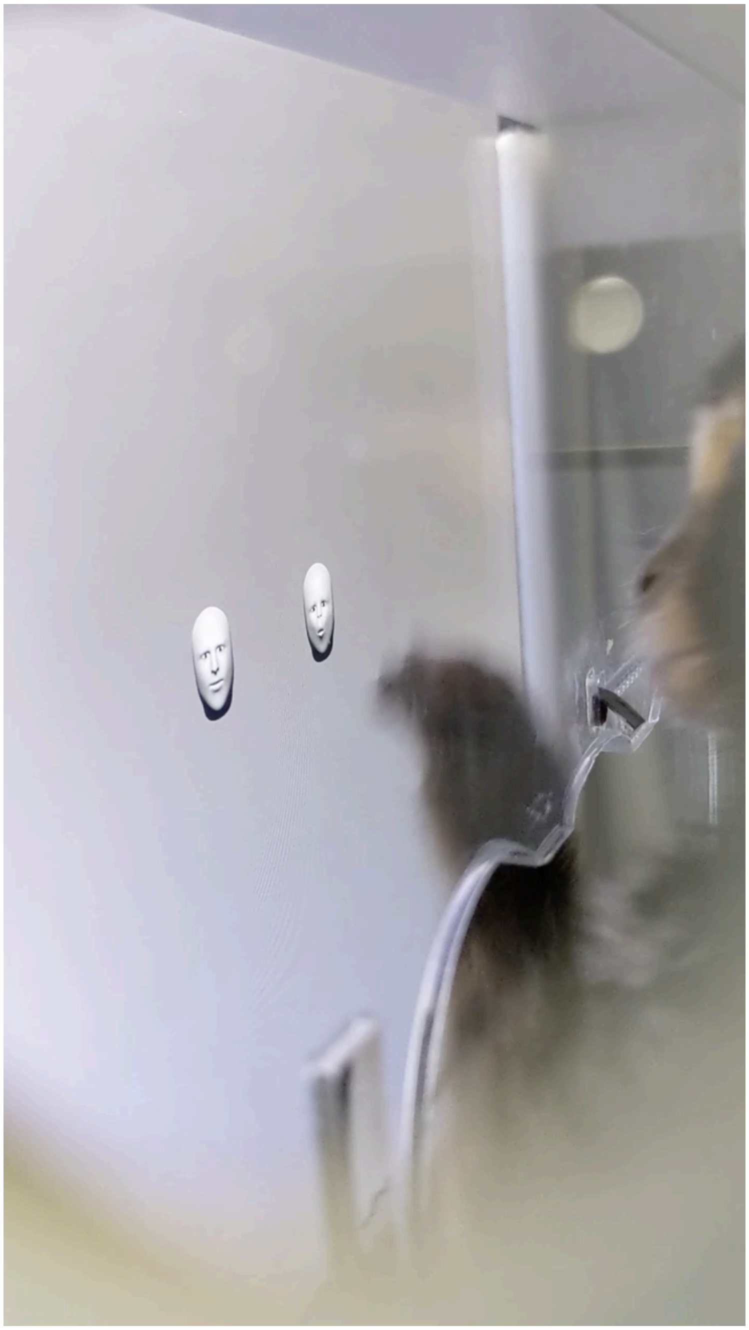

Video S1. A marmoset subject performing an early version of the face discrimination task on a touchscreen device in their homecage.
